# Prospective evaluation of Ki-67 system in histological grading of soft tissue sarcomas in the Japan Clinical Oncology Group Study JCOG0304

**DOI:** 10.1186/s12957-016-0869-6

**Published:** 2016-04-18

**Authors:** Kazuhiro Tanaka, Tadashi Hasegawa, Takayuki Nojima, Yoshinao Oda, Junki Mizusawa, Haruhiko Fukuda, Yukihide Iwamoto

**Affiliations:** Department of Endoprosthetic Surgery, Oita University, Yufu, Oita 879-5593 Japan; Department of Surgical Pathology, Sapporo Medical University School of Medicine, South 1 West 16, Chuo-ku, Sapporo, 060-8543 Japan; Department of Pathology and Laboratory Medicine, Kanazawa Medical University, Ishikawa, 920-0265 Japan; Department of Anatomic Pathology, Kyushu University, Fukuoka, 812-8582 Japan; JCOG Data Center, National Cancer Center, Tokyo, 104-0045 Japan; Department of Orthopaedic Surgery, Kyushu University, Fukuoka, 812-8582 Japan

**Keywords:** Ki-67, Mitosis, Prospective study, Sarcoma, Tumor grading

## Abstract

**Background:**

The correct clinical staging of soft tissue sarcomas (STS) is critical for the selection of treatments. The staging system consists of histological grade of the tumors and French Federation of Cancer Center (FNCLCC) system based on mitotic count is widely used for the grading. In this study, we compared the validity and usefulness of Ki-67 grading system with FNCLCC system in JCOG0304 trial which investigated the efficacy and safety of perioperative chemotherapy with doxorubicin and ifosfamide for STS.

**Methods:**

All 70 eligible patients with STS in the extremities treated by perioperative chemotherapy in JCOG0304 were analyzed. Univariate and multivariate Cox regression analyses were conducted to investigate an influence on overall survival.

**Results:**

The reproducibility of Ki-67 grading system in the histological grading of STS was higher than FNCLCC system (*κ* = 0.54 [95 % CI 0.39–0.71], and 0.46 [0.32–0.62], respectively). Although FNCLCC grade was not associated with overall survival (OS) in univariate analysis (HR 2.80 [0.74–10.55], *p* = 0.13), Ki-67 grading system had a tendency to associate with OS in univariate analysis (HR 4.12 [0.89–19.09], *p* = 0.07) and multivariate analysis with backward elimination (HR 3.51 [0.75–16.36], *p* = 0.11).

**Conclusions:**

This is the first report demonstrating the efficacy of Ki-67 grading system for the patients with STS in the prospective trial. The results indicate that Ki-67 grading system might be useful for the evaluation of histological grade of STS.

## Background

Soft tissue sarcomas (STS) in adults are rare malignant tumors, and the incidence of STS is approximately 1 % of all malignant tumors. According to the Soft Tissue Tumor Registry reported by the Musculoskeletal Tumor Committee of the Japanese Orthopaedic Association, only 1540 cases of STS were registered in 2012 in Japan [1].

The prognosis and standard treatments of STS differ in the clinical stages of the tumor. The American Joint Committee on Cancer (AJCC)/International Union against Cancer (UICC) staging system is the most widely used for the staging of STS [2]. Surgical resection of the tumor with or without radiotherapy is the standard treatment and highly successful for stage I and II STS, and systemic chemotherapy with doxorubicin (DOX)-based regimen is standard for stage IV STS [3]. The standard therapeutic modality for stage III STS is mainly surgical resection; however, systemic perioperative chemotherapy with DOX plus ifosfamide (IFO) is also the promising treatment for high-risk STS [4]. Therefore, the precise diagnosis and staging is critical for the selection of treatments and improvement of outcome of the patients with STS.

The clinical staging system is based on the histological grade of STS. It has been shown that the histological grade correlates well with prognosis of the patients with STS [5]. Various histological grading systems of STS have been proposed. Among them, French Federation of Cancer Center (FNCLCC) system is one of the most popular and widely used grading systems [6]. In the FNCLCC system, histological grading is rated by the total of the scores for three parameters, including the tumor differentiation, degree of necrosis, and mitotic count. However, the mitotic count is affected by various factors such as the interval between surgical resection and fixation of tumor tissue, cell size and tumor cellularity, and the experience of the pathologists [7–10], so the development of more precise and objective grading system has been required.

Recently, the usefulness of Ki-67 (MIB-1) score has been reported in some retrospective studies. Using the retrospective data of 95 patients with STS of the extremities, trunk, head, and neck treated in the single institution, we have previously reported the usefulness of a novel histological grading system based on the three parameters: tumor differentiation, degree of necrosis, and Ki-67 (MIB-1) score [7]. In this system, the mitotic count in FNCLCC system was replaced by cell count in Ki-67 immunohistochemical staining. We have shown that the Ki-67 grading system was the most significant independent prognostic factor for the patients with STS in the multivariate analysis [11]. We also retrospectively analyzed validity (sensitivity and specificity) and reproducibility (agreement) of diagnosis of histological grade using Ki-67 grading and FNCLCC systems for STS by comparing the independent diagnosis of four pathologists and the gold standard which was diagnosed by two experts who developed Ki-67 grading system [12]. The results indicated that the validity and reproducibility of Ki-67 grading system in the diagnosis of histological grading of STS was higher than that of FNCLCC system.

We have conducted the phase II clinical trial for STS, Japan Clinical Oncology Group Study JCOG0304. In JCOG0304, patients with operable, high-grade STS in the extremities were treated with perioperative DOX 60 mg/m^2^ plus IFO 10 g/m^2^ for three courses with 3-week interval, followed by operation and postoperative two courses of the same regimen [13, 14]. To further evaluate the validity and reproducibility of Ki-67 grading system in prospective study, we compared Ki-67 grading system with FNCLCC system using the clinical data of the patients in JCOG0304. We also analyzed the factors including both Ki-67 and FNCLCC grades that might influence on survival of the patients treated in JCOG0304.

## Methods

### Patients

Records of 72 patients with STS enrolled in JCOG0304 trial conducted by JCOG Bone and Soft Tissue Tumor Study Group (JCOG-BSTTSG) were used in the present study. Details of eligibility criteria in JCOG0304 have been stated elsewhere [14]. Key inclusion criteria of the trial was as follows: (1) a histological diagnosis of STS as undifferentiated pleomorphic sarcoma, fibrosarcoma, leiomyosarcoma, synovial sarcoma, liposarcoma, pleomorphic rhabdomyosarcoma, or undifferentiated sarcoma using open biopsy specimen; (2) FNCLCC histological grading system: grade 2 or 3; (3) AJCC/UICC (6th edition) stage III (T2bN0M0); (4) resectable tumor in the extremities; (5) measurable lesion on magnetic resonance imaging (MRI) axial section; (6) age between 20 and 70 years; (7) Eastern Cooperative Oncology Group (ECOG) performance status 0 or 1; and (8) sufficient organ function.

The patients were treated with preoperative chemotherapy consisted of DOX (30 mg/m^2^/day, days 1 and 2) and IFO (2 g/m^2^/day, days 1 to 5), and was repeated for three courses every 3 weeks. The tumor was resected within 5 weeks of the last course of preoperative chemotherapy. When the tumor resection was completed, two courses of the same regimen as preoperative chemotherapy with DOX and IFO were carried out every 3 weeks. No additional therapy was given until the patient exhibited treatment failure including local recurrence and/or distant metastasis.

The study protocol was approved by the Clinical Trial Review Committee of JCOG, and also approved by the Institutional Review Boards of each of the 27 participating institutes. All patients gave written informed consent before entry to the study.

### Pathological central review

Histological diagnosis and grading of the biopsy samples from all patients in the present study were reviewed by the Central Pathological Committee of JCOG-BSTTSG. To obtain enough amounts of tumors, all samples were collected by open biopsy. Needle biopsy was not allowed in the present study. The committee consisted of three pathologists specialized in diagnosis of STS from three institutions (TH, YO, and TN). The review was independently performed by each pathologist; then, the consensus diagnosis of the tumor was determined by the committee meeting.

Ki-67 immunostaining was carried out by the Central Pathological Committee for the grading of all tumor samples as previously described [7]. Briefly, 4-μm-thick sections were stained with antibodies for Ki-67 (clone: MIB-1, 1:100 dilution, DAKO, Tokyo, Japan). The sections were subjected to heat-induced epitope unmasking with Target Retrieval Solution (pH 9, DAKO) using microwave for 20 min. Ki-67 score was assessed by counting the percentages of Ki-67-positive nuclei per 1000 tumor cells in the region of the tumor with the greater density of staining, which usually corresponded to the areas with the highest mitotic activity in the tumor. The histologic grade was calculated by adding scores of three factors; tumor differentiation, tumor necrosis, and Ki-67 immunostaining, each of which was given a score of 0 to 3. Thus, in this system, the mitotic count in FNCLCC system was simply replaced by cell count in Ki-67 immunostaining. A Ki-67 score of 1 was assigned to lesions with 0–9 % of the tumor cells positive for Ki-67 immunostaining, a score of 2 was those with 10–29 %, and a score of 3 was those with ≥30 % of the tumor cells positive for Ki-67 immunostaining, respectively. The standard FNCLCC grading system was also used in this study.

### Data management and treatment evaluation

The JCOG Data Center performed data management and statistical analysis. The center also performed central monitoring to ensure data submission, patient eligibility, protocol compliance, safety, and on-schedule study progress. None of the orthopedic surgeons who performed the protocol treatment were involved in the data analysis.

### Statistical method

As a measure of reproducibility, weighted kappa statistics (*κ*) with Cicchetti-Allison weight type [15] in each pair of the three pathologists, i.e., three combinations, was calculated both for FNCLCC and Ki-67 grading systems. Confidence interval of weighted kappa statistics for overall agreement between pairs of the pathologists was estimated by bootstrap sampling method [16]. As a measure of agreement and reproducibility, *κ* value is commonly interpreted as follows: 0.00–0.20, slight; 0.21–0.40, fair; 0.41–0.60, moderate; 0.61–0.80, substantial; and 0.81–1.00, almost perfect agreement [17].

Overall survival (OS) was defined as the time from enrollment to death and censored at the date of last contact for a surviving patient. The overall survival was estimated by Kaplan-Meier method.

Univariate and multivariate Cox regression analysis was performed to investigate the impact on overall survival. Hazard ratios and *p* values were derived by Cox regression model. The following factors were investigated: age, sex, ECOG performance status, tumor location, tumor size, histological subtype, tumor differentiation score, tumor necrosis score, tumor mitosis score, histological grade assessed by FNCLCC system, and histological grade assessed by Ki-67 grading system. Of these, data retrieved by institutional decision was used only for univariate analysis, while the data reviewed by the Central Pathological Committee was used both for univariate and multivariate analyses. As a sensitivity analysis, multivariate analysis with backward elimination method with alpha of 0.2 was also performed. Statistical analysis was done with SAS version 9.1 or more (SAS Institute, Cary, NC).

## Results

### Patient characteristics

From March 2004 to September 2008, a total of 72 patients were enrolled into the JCOG0304 trial, and 70 eligible patients in the trial were included in the present analysis.

The characteristics of the patients were as follows. Briefly, 36 patients were male and 34 patients were female, and the median age of patients was 48.5 years old (range 21–66 years). Tumor location included the thigh in 34 patients, the calf in 9 patients, the other sites of the lower extremity in 14 patients, the shoulder in 6 patients, the upper arm in 2 patients, the forearm in 1 patient, and the other sites of the upper extremity in 4 patients. The median tumor size was 7.45 cm. The histological diagnosis of tumors by institutional decision was as follows: synovial sarcoma in 20 patients, undifferentiated pleomorphic sarcoma in 17 patients, leiomyosarcoma in 11 patients, fibrosarcoma in 5 patients, liposarcoma in 4 patients, undifferentiated sarcoma in 4 patients, pleomorphic rhabdomyosarcoma in 2 patients, and other histological subtype in 7 patients.

### Histological grading using Ki-67 and mitosis

**Fig. 1:**
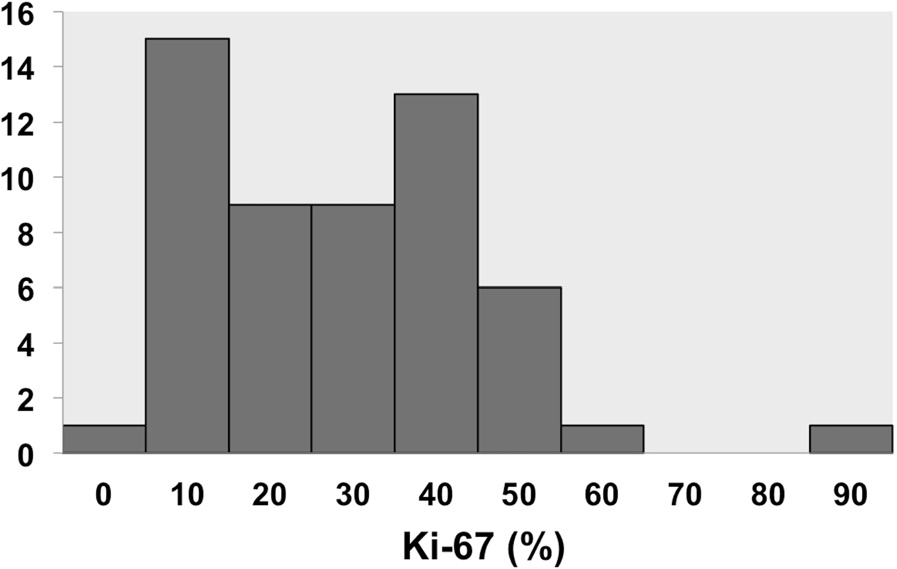
Distribution of Ki-67 immunostaining in JCOG0304 study

Among 70 tumors, according to the grading system using mitosis, 36 and 34 tumors were assessed as grade 2 and grade 3, respectively, by the Pathological Central Committee. On the other hand, with the grading system using Ki-67 immunostaining, 32 and 38 tumors were assessed as grade 2 and grade 3, respectively (Table 1). Seven tumors assessed as grade 2 using mitosis were evaluated as grade 3 using Ki-67, whereas three tumors assessed as grade 3 using mitosis were evaluated grade 2 using Ki-67, respectively. The distribution of Ki-67 immunostaining ranged from 1 to 90 % (median 25 %) (Fig. 1). The distribution pattern was similar to that in the previous report [17].

**Table 1:** Histological grading by Ki-67 immunostaining and mitotic count (*n* = 70)

		Grade using Ki-67		
		Grade 2	Grade 3	Total
Grading using mitosis	Grade 2	29	7	36
	Grade 3	3	31	34
	Total	32	38	70

Next, the reproducibility of each grading system was evaluated. Of 70 tumors, 67 tumors were evaluated for the analysis of the reproducibility. The agreement between pathologist 1 and 2, 1 and 3, and 2 and 3 for Ki-67 immunostaining was *κ* = 0.65 (95 % CI 0.47–0.83), 0.54 (95 % CI 0.34–0.73), and 0.41 (95 % CI 0.20–0.61), respectively (Table 2). On the other hand, the agreement between pathologist 1 and 2, 1 and 3, and 2 and 3 for mitosis was *κ* = 0.45 (95 % CI 0.26–0.65), 0.47 (95 % CI 0.27–0.67), and 0.47 (95 % CI 0.26–0.67), respectively (Table 3). Taken together, the overall agreement between pairs of the pathologists for Ki-67 was *κ* = 0.54 (95 % CI 0.39–0.71), and for mitosis was *κ* = 0.46 (95 % CI 0.32–0.62).

**Table 2:** Reproducibility of grading using Ki-67 (*n* = 67)

		Pathologist 2			
		Grade 1	Grade 2	Grade 3	Total
Pathologist 1	Grade 1	2	0	0	2
	Grade 2	0	22	8	30
	Grade 3	0	5	30	35
	Total	2	27	38	67
	*κ* = 0.65 (95 % CI 0.47–0.83)				
		Pathologist 3			
Pathologist 1	Grade 1	1	1	0	2
	Grade 2	0	25	5	30
	Grade 3	0	11	24	35
	Total	1	37	29	67
	*κ* = 0.54 (95 % CI 0.34–0.73)				
		Pathologist 3			
Pathologist 2	Grade 1	1	1	0	2
	Grade 2	0	21	6	27
	Grade 3	0	15	23	38
	Total	1	37	29	67
	*κ* = 0.41 (95 % CI 0.20–0.61)

**Table 3:** Reproducibility of grading using mitosis (*n* = 67)

		Pathologist 2			
		Grade 1	Grade 2	Grade 3	Total
Pathologist 1	Grade 1	0	1	0	1
	Grade 2	2	30	9	41
	Grade 3	0	7	18	25
	Total	2	38	27	67
	*κ* = 0.45 (95 % CI 0.26–0.65)				
		Pathologist 3			
Pathologist 1	Grade 1	0	1	0	1
	Grade 2	2	32	7	41
	Grade 3	0	8	17	25
	Total	2	41	24	67
	*κ* = 0.47 (95 % CI 0.27–0.67)				
		Pathologist 3			
Pathologist 2	Grade 1	1	1	0	2
	Grade 2	1	30	7	38
	Grade 3	0	10	17	27
	Total	2	41	24	67
	*κ* = 0.47 (95 % CI 0.26–0.67)				

### Prognostic factors

**Fig. 2:**
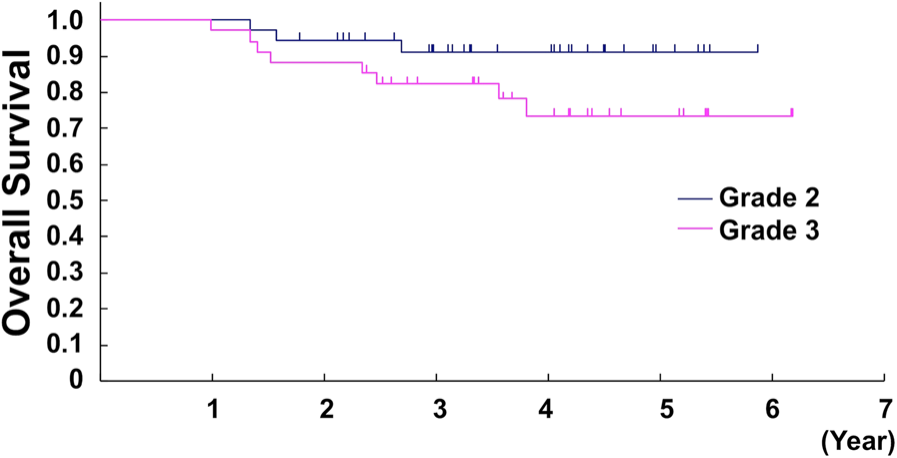
Kaplan-Meier estimates of overall survival by FNCLCC grading (*n* = 70)

**Fig. 3:**
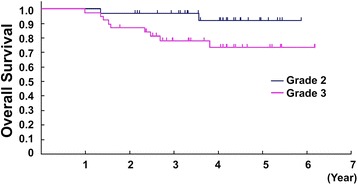
Kaplan-Meier estimates of overall survival by Ki-67 grading (*n* = 70)

With a median follow-up of 3.6 years for 70 eligible patients included in this study, the proportion of 2-year and 5-year OS for the included patients in the present analysis (*n* = 70) was 91.4 % (95 % CI, 81.9–96.1 %) and 81.8 % (95 % CI, 68.9–89.7 %), respectively. There were no significant differences in survival regarding age (<50 vs. ≥50), sex (male vs. female), tumor size (<10 vs. ≥10 cm, or <8 vs. ≥8 cm), histological subtypes (UPS vs. synovial sarcoma vs. others), histological tumor differentiation (score 2 vs. 3), tumor necrosis (score 2 vs. 3), and mitosis (score 1 vs. 2 vs. 3) (Table 4). There was also no significant difference between survival of the patients with FNCLCC grade 2 tumors (5-year OS 90.9 %, [95 % CI 74.3–97.0]) and that with grade 3 tumors (5-year OS 73.5 %, [95 % CI 53.2–86.1]) (HR 2.80, 95 % CI 0.74–10.55, *p* = 0.13) (Fig. 2). However, survival of the patients with the tumor assessed as histologic grade 3 by Ki-67 immunostaining was worse than that with the tumor assessed as grade 2. The 5-year OS for grade 3 and grade 2 tumors using Ki-67 was 73.5 % (95 % CI 54.4–85.6) (*n* = 38) and 91.8 % (95 % CI 69.8–98.0) (*n* = 32), respectively (HR 4.12, 95 % CI 0.89–19.09, *p* = 0.070) (Fig. 3, Table 4). While FNCLCC grading system was not selected as a prognostic factor by multivariate analysis with backward elimination, Ki-67 grading system was also tended to associate with OS on multivariate analysis with backward elimination (HR 3.51, 95 % CI 0.75–16.36, *p* = 0.11) (Table 5).

**Table 4:** Univariate analysis for survival

Factors	Category	Univariate analysis HR (95 % CI)	
			*p* value
Sex	Female (vs. male)	0.89 (0.27–2.93)	0.85
Age (years)	≥40 (vs. <40)	5.12 (0.65–40.06)	0.12
Performance status	1 (vs. 0)	0.59 (0.13–2.72)	0.50
Histological subtype	Leiomyosarcoma (vs. UPS)	0.39 (0.04–3.51)	0.40
	Synovial sarcoma (vs. UPS)	0.69 (0.15–3.08)	0.63
	Others (vs. UPS)	0.64 (0.14–2.87)	0.56
Tumor differentiation	3 (vs. 2)	0.71 (0.22–2.31)	0.56
Tumor necrosis	1 or 2 (vs. 0)	2.96 (0.64–13.73)	0.16
Tumor mitosis	2 or 3 (vs. 1)	1.28 (0.37–4.36)	0.70
Histological grade (FNCLCC)	Grade 3 (vs. 2)	2.80 (0.74–10.55)	0.13
Histological grade (Ki-67)	Grade 3 (vs. 2)	4.12 (0.89–19.09)	0.070

**Table 5:** Multivariate analysis for survival

Factors	Category	Multivariate analysis (including all variables) HR (95 % CI)	
			*p* value
Sex	Female (vs. male)	1.75 (0.49–6.30)	0.39
Age (years)	≥40 (vs. <40)	8.25 (0.85–79.91)	0.069
Performance status	1 (vs. 0)	0.60 (0.12–2.95)	0.53
Histological subtype	Leiomyosarcoma (vs. UPS)	0.29 (0.03–2.85)	0.29
	Synovial sarcoma (vs. UPS)	1.42 (0.27–7.47)	0.68
	Others (vs. UPS)	0.55 (0.12–2.60)	0.45
Histological grade (FNCLCC)	Grade 3 (vs. 2)	2.24 (0.35–14.30)	0.39
Histological grade (Ki-67)	Grade 3 (vs. 2)	2.79 (0.32–24.41)	0.35
Factors	Category	Multivariate analysis (backward elimination with alpha = 20 %) HR (95 % CI)	
			*p* value
Sex	Female (vs. male)		
Age (years)	≥40 (vs. <40)	4.21 (0.53–33.28)	0.17
Performance status	1 (vs. 0)		
Histological subtype	Leiomyosarcoma (vs. UPS)		
	Synovial sarcoma (vs. UPS)		
	Others (vs. UPS)		
Histological grade (FNCLCC)	Grade 3 (vs. 2)		
Histological grade (Ki-67)	Grade 3 (vs. 2)	3.51 (0.75–16.36)	0.11

## Discussion

In the present study, we investigated the differences in the diagnosis of the histological grade of STS assessed by Ki-67 expression levels and mitosis using the biopsy samples of STS in JCOG0304. We further analyzed the impact of prognostic factors including the histological grade on survival of the patients with STS in the clinical trial. The results demonstrated that there was the substantial disagreement (14.3 %) between Ki-67 grading system and FNCLCC system, and that Ki-67 grading system might exhibit better reproducibility in the assessment of histological grading of STS in the extremities than mitotic score. Potential prognostic value of Ki-67 has also been shown.

We have previously demonstrated that the grading system using Ki-67 immunostaining was better in terms of the validity and reproducibility than FNCLCC system using mitotic score [12]. We have also indicated that the Ki-67 grading system was significantly associated with the prognosis of the patients with STS in the multivariate analysis [11]. However, those reports were retrospective analyses from a single institution. Therefore, we investigated the validity and reproducibility of the Ki-67 grading system in the grading of STS treated by the same regimen in the multi-institutional clinical trial, JCOG0304 [13, 14].

In the present study, the histological grade of 10 tumors among 70 (14.3 %) showed disconcordance between FNCLCC system and Ki-67 grading system, indicating that the disagreement between both systems was not negligible. Thus, the reproducibility was calculated as kappa statistics to elucidate which system would be better. The averaged weighted kappa statistics between pairs of three expert pathologists for Ki-67 was *κ* = 0.54 (95 % CI 0.39–0.71), and for mitosis was *κ* = 0.46 (95 % CI 0.32–0.62). In the retrospective study, the *κ* score was 0.61 for Ki-67 immunostaining and 0.54 for mitotic score, suggesting the superiority of Ki-67 grading system to FNCLCC system [9]. It was also reported that the *κ* statistics using mitosis of FNCLCC system was 0.38 for FNCLCC grade 2 and 0.48 for grade 3 tumors, respectively [18]. The possible reason for the better reproducibility of Ki-67 grading system over FNCLCC system is that Ki-67 is expressed in all phases of the cycle except G0 and is a better measure of dividing cells than HE staining [19]. Our results were consistent with those in the previous reports, and demonstrating that Ki-67 grading system is also valid in the prospective study. On the other hand, Ki-67 grading system depends on the immunoreactivity of Ki-67 antigen in the tumor specimens. Thus, the results of Ki-67 scoring might be affected by various factors during immunohistochemical study including fixation time and evaluation methods. To minimize the influence of these factors on Ki-67 immunostaining, we standardized the immunohistochemical procedure and all staining were carried out only in one institute (Kyushu University). However, there is a possibility that the results of the present study might be affected by the variation of Ki-67 immunostaining among specimens.

The results in the present study also exhibited a substantial disagreement of tumor grading using Ki-67 and mitosis. It has been reported that overall percent agreement of Ki-67 and mitosis grading among four pathologists was reported to be 79 % (95 % CI, 76–83) and 69 % (95 % CI, 65–73) [12]. These observations demonstrated an obvious limitation in the current method for assessment of tumor grading even with Ki-67 system by manual counting of Ki-67 immunostaining. Further improvement such as measurement by a digital image analysis system for Ki-67 assessment should be needed to overcome the difficulties in correct evaluation of histological grade of STS.

Regarding the prognostic relevance, there was a tendency that the Ki-67 grading system was the potential prognostic factor associated with OS of the patients in the present study. However, the difference of OS in the present study was smaller than that in the previous report [11]. One of the possible reasons for this discrepancy is that the outcome of JCOG0304 was far better than expected. An Italian randomized study for STS in the extremities has demonstrated that 4-year OS of the patients treated by adjuvant chemotherapy with epirubicin plus IFO was 69 % (95 % CI, 68.9–89.7 %) [20]. On the other hand, 5-year OS for eligible patients was 81.8 % in JCOG0304. Furthermore, our retrospective study has been reported that 5-year OS for grade 2 and grade 3 STS was 71.8 and 44.3 %, respectively [11]. In JCOG0304, 5-year OS for grade 2 and grade 3 STS was 91.8 and 73.5 %, respectively. The results suggested that the prognosis of the patients with STS in JCOG0304 was remarkably improved by the intensive pre- and postoperative chemotherapy with DOX and IFO. The survival of the patients with grade 3 tumors in the present study was comparable to that with grade 2 tumors in the previous report. The high survival rate of the patients with grade 3 tumors in this trial might be one of the reasons why the difference of the survival between grade 2 and grade 3 tumors was not statistically significant. Since the trial was phase II study, JCOG0304 had selection biases including many patients with good prognosis histologic subtypes of the tumors, and the majority of grade 2 tumor in FNCLCC grading (47 patients out of 72). Thus, there is a possibility that these imbalanced factors might have affected to the results of the present study.

In summary, it is suggested that Ki-67 grading system might show better reproducibility and validity in the assessment of histological grade for STS in the extremities than FNCLCC system. Furthermore, among factors tested in the present study, there was a tendency that Ki-67 grading system was associated with survival in univariate analysis. Ki-67 grade was also suggested as a candidate of prognostic factor in multivariate analysis with backward elimination.

## Conclusions

This is the first report demonstrating the efficacy of Ki-67 grading system for the patients with STS in the prospective trial. The results indicate that Ki-67 grading system might be useful for the evaluation of histological grade of STS.
